# Diet quality in young adulthood and sleep at midlife: a prospective analysis in the Bogalusa Heart Study

**DOI:** 10.21203/rs.3.rs-3788358/v1

**Published:** 2023-12-27

**Authors:** Kaitlin S. Potts, Jeanette Gustat, Maeve Wallace, Sylvia Ley, Lu Qi, Lydia A. Bazzano

**Affiliations:** Tulane University School of Public Health and Tropical Medicine; Tulane University School of Public Health and Tropical Medicine; Tulane University School of Public Health and Tropical Medicine; Tulane University School of Public Health and Tropical Medicine; Tulane University School of Public Health and Tropical Medicine; Tulane University School of Public Health and Tropical Medicine

**Keywords:** diet quality, dietary patterns, insomnia, sleep apnea, sleep quality

## Abstract

**Background:**

Diet and sleep are both established risk factors for cardiometabolic diseases. Prior evidence suggests a potential link between these behaviors, though evidence for how they associate with each is scarce. This study aimed to determine the association between diet quality in young adulthood and multiple sleep outcomes at midlife in the Bogalusa Heart Study (BHS).

**Methods:**

This prospective study included 593 BHS subjects with dietary assessment at the 2001–2002 visit and sleep questionnaire responses from the 2013–2016 visit, after an average of 12.7 years (baseline mean age: 36 years, 36% male, 70%/30% White and Black persons). A culturally tailored, validated food frequency questionnaire assessed usual diet. Diet quality was measured with the Alternate Healthy Eating Index (AHEI) 2010, the Healthy Eating Index (HEI) 2015, and the alternate Mediterranean (aMed) dietary score. Robust Poisson regression with log-link function estimated risk ratios (RR) for insomnia symptoms, high sleep apnea score, and having a healthy sleep pattern by quintile and per standard deviation (SD) increase in dietary patterns. Models adjusted for potential confounders including multi-level socioeconomic factors, depression, and body mass index. Trends across quintiles and effect modification by sex, race, and education were tested.

**Results:**

Higher diet quality in young adulthood, measured by both AHEI and HEI, was associated with lower risk of having insomnia symptoms at midlife. In the adjusted model, each SD-increase in AHEI (7.8 points; 7% of score range) conferred 15% lower risk of insomnia symptoms at follow-up (RR [95% confidence interval CI]: 0.85 [0.77, 0.93]), those in Q5 of AHEI had 0.54 times the risk as those in Q1 (95% CI: 0.39, 0.75), and there was a significant decreasing risk trend across quintiles (trend p = 0.001). There were no significant associations between young adult diet quality and having a high sleep apnea risk or a healthy sleep pattern at follow-up.

**Conclusions:**

A healthy diet was associated with a lower risk of future insomnia symptoms. If replicated, these findings could have implications for chronic disease prevention strategies incorporating the lifestyle behaviors of sleep and diet.

## Background

Poor diet quality is a known risk factor for multiple chronic diseases including cardiovascular disease (CVD), type-2 diabetes, and cancer as well as all-cause mortality. Higher adherence to the Alternate Healthy Eating Index (AHEI), a dietary pattern assessing overall diet quality, conferred 21% risk reduction for CVD in a recent pooled analysis of the Nurses’ Health Study and the Health Professionals Follow-up Study (1). However, the majority of Americans fall short of healthy eating recommendations (2). Similarly, meta-analyses show that short sleep duration, insomnia, and sleep apnea increase risk of CVD and type-2 diabetes (3). Estimates suggest that at least 30% of Americans suffer from short sleep (less than 7 hours), insomnia, and moderate to severe sleep apnea (4–6).

Despite the known effects of diet and sleep on disease outcomes, evidence for the influence of diet on sleep is limited, especially in long-term community cohorts. If diet is an important contributor to sleep health, clarifying this relationship could have implications for improving the effectiveness of programs aiming to reduce chronic disease risk. Some experimental studies have identified relationships between certain foods (e.g. milk, oysters, salmon, and kiwi fruit) and sleep quality and duration in the short-term (7–13). A few observational studies have looked at associations between overall diet quality with sleep cross-sectionally, generally finding that higher diet quality associates with better sleep outcomes (14–20). Fewer studies have assessed prospective associations between diet quality on sleep outcomes in observational cohorts (16, 21, 22). They found that higher adherence to a Mediterranean-type diet was associated with better sleep quality, higher sleep efficiency, and fewer insomnia symptoms. However, these studies only used a Mediterranean dietary pattern to assess diet quality, did not simultaneously assess multiple domains of sleep, and none investigated an association with sleep apnea. These studies were conducted in an elderly Spanish cohort, an all women cohort, and the Multi-Ethnic Study of Atherosclerosis (MESA), an older U.S. cohort based in urban centers. Thus, evidence is sparse for the effect of diet quality on multiple sleep health outcomes from prospective cohort analyses, using measures of diet quality beyond a Mediterranean diet, and that are relevant for lower-income, younger, non-urban U.S. communities.

The aim of this study was to assess the associations between diet quality in early adulthood with sleep outcomes at midlife in the Bogalusa Heart Study (BHS) cohort and to determine if differences in these relationships were present by sex, race, or socioeconomic status. The hypothesis was that higher diet quality in early adulthood confers risk protection against adverse sleep outcomes.

## Methods

### Study design and population

The BHS began in 1972 as a series of cross-sectional surveys in the semi-rural community of Bogalusa in southeastern Louisiana and has continued to re-examine participants to the present day (23). Study procedures were approved by the Tulane University Health Sciences Institutional Review Board and all participants gave written, informed consent at each visit. Among 1203 participants in the 2001–2002 BHS exam, dietary assessment was completed on 1186 and 22 of these were excluded for implausible energy intake (< 500 or > 5000 kcal/day (24); see **Figure S1**, Additional file 1 for participant flowchart). Of the 1164 with baseline diet data, 656 completed a sleep questionnaire at the 2013–2016 follow-up visit. Participants were excluded for having a history of heart attack at baseline (n = 12) or missing baseline covariate information (n = 51; 38 for residential addresses that could not be geocoded, e.g. P.O. boxes, 13 for other missing covariates). There were 593 subjects included in this analysis with an average follow-up time of 12.7 years.

### Measurement of diet quality

Diet was measured in the 2001–2002 visit with the Youth/Adolescent Questionnaire (YAQ), a 151-item semiquantitative food frequency questionnaire (FFQ) adapted from the Nurses Health Initiative FFQ for younger populations by including more relevant snack foods (e.g. pop tarts and Jell-O) and by designing it to be easier to complete (25). The YAQ asks about typical frequency of consumption of food items over the past year and uses natural portion sizes (e.g., one slice of bread, a sandwich, a glass of milk). Although BHS participants were young adults at this visit, no other validated FFQ available at the time was more appropriate for the population. The YAQ was designed for use in a general US population and was tested for reproducibility and validity among youths in different communities from 20 states (24, 25). The reproducibility of the YAQ was established by repeated administration of the questionnaire one year apart among a multiethnic youth population in 1993–94 (26). The mean Pearson correlation between the one-year apart measurements across several different nutrients was 0.55 and the mean across food groups was 0.49. The relative validity of the YAQ was determined by comparing average intakes of nutrients from two YAQs to three 24-hour dietary recalls and the average correlation coefficient across the nutrients tested was 0.54 (24). These measures of reproducibility and validity are in alignment with other measures of dietary intake, especially FFQs (27). In addition, the estimates for validity are thought to be attenuated by the impact of day-to-day variability on the comparator method, 24-hour recalls.

Nutrient intakes were estimated by the Channing Laboratory at Harvard University, the developers of the YAQ (28). Intake of food groups were obtained by matching the YAQ foods to the USDA Food Patterns Equivalent Database (FPED) (29). Nutrient and food group intakes were used to calculate three dietary pattern scores: Healthy Eating Index 2015 (HEI-2015), Alternate Healthy Eating Index 2010 (AHEI-2010), and Alternate Mediterranean diet score (aMed). The HEI-2015 includes 13 components measuring adherence to the 2015 Dietary Guidelines for Americans where a higher score (range 0 to 100) indicates closer adherence (30). The AHEI-2010 directly incorporates scientific evidence of the relationship between diet and health and includes 11 components worth 10 points each (31). The AHEI-2010 used in this study was modified to be a 10-component score (range 0–100) since trans-fats were not available from the original nutrient analysis. The aMed (9 components, range 0–9) measures a Mediterranean-type diet emphasizing plant foods, monounsaturated fats, and fish while discouraging intakes of saturated fats and animal foods (32). See Supplementary **Table S1**, Additional File 1 for detailed scoring of each dietary pattern.

### Measurement of sleep outcomes

The 2013–2016 BHS study visit included a sleep questionnaire with multiple validated instruments. The Women’s Health Initiative Insomnia rating Scale (WHIIRS) is a 5-item scale asking the frequency common insomnia symptoms: trouble falling to sleep, night waking, waking too early, trouble falling back to sleep, and overall sleep quality (see Table S2, Additional file 1 for detailed questions). A score > 9 is a valid and reliable indicator that someone has a high risk of insomnia in comparison to several objective measures (33, 34). Sleep apnea was measured with the Berlin Questionnaire, a validated instrument assessing snoring, sleepiness, and presence of obesity or hypertension (see Table S3, Additional file 1) (35). One is considered to have a high risk for sleep apnea when they score positive on two of the three domains. This classification was validated with 86% sensitivity and 77% specificity to correspond to a clinical indication of mild sleep apnea, measured objectively by apnea-hypopnea index > 5 (35). An overall measure of healthy sleep -- the healthy sleep pattern- was assessed similarly to the method used by Fan et al., who showed a healthy sleep pattern was associated with lower CVD risk in the UK Biobank and the China Kadoorie Biobank (36). The healthy sleep pattern was dichotomized to identify individuals scoring healthy on four or more of five sleep domains. Healthy for each domain included identifying as a morning chronotype, typically sleeping 7–8 hours, and reporting infrequent insomnia symptoms, snoring, and daytime sleepiness. The reduced Morningness-Eveningness Questionnaire (MEQ) assessed chronotype, the Epworth Sleepiness Scale assessed daytime sleepiness, the WHIIRS was used to identify insomnia symptoms, and snoring was assessed with the snoring component of the Berlin Questionnaire (34, 35, 37, 38).

### Covariates

Demographic characteristics were assessed at the 2001–2002 baseline visit. Self-rated physical activity at work and leisure time were measured with validated questionnaires (39). These physical activity measures were also validated in the BHS via correlation with metabolic syndrome (40). The Centers for Epidemiologic Studies Depression scale (CES-D) assessed depressive symptoms (41). Body mass index (BMI), weight in kilograms/height in meters^2^, used average weight and height of two measures and waist circumference was measured in triplicate.

To further capture socioeconomic and neighborhood contextual factors, residential addresses were geocoded to obtain census tracts and incorporate 2000 Decennial Census data. The Index of Concentration at the Extremes (ICE) was calculated as a measure of segregation based on income and race (42). The ICE was calculated in each tract as the number of White householders reporting ≥$100,000 annual income minus the number of Black householders reporting <$25,000 annual income, divided by the total number of households reporting income in the tract. The ICE ranges from − 1 to 1, where a negative ICE indicates more members of the disadvantaged group relative to the privileged group in the area.

### Statistical analysis

Participants were grouped into quintiles of dietary pattern scores to assess non-linearity and minimize the influence of outliers, in addition to evaluating the diet scores as continuous variables. Means and standard deviations (SD) for continuous variables or frequency (percentage) for categorical variables were calculated to describe the total sample and per quintile of AHEI-2010. Differences across AHEI-2010 quintiles were tested for with ANOVA or Pearson chi-squared tests. Robust Poisson regression models with a log-link function were used to estimate risk ratios (RR) for insomnia symptoms (WHIIRS > 9), high sleep apnea score (positive on Berlin Questionnaire), and having a healthy sleep pattern at follow-up by quintile (using Q1 as reference) and per SD increase in baseline dietary pattern scores. Trends across quintiles were tested by assigning the median dietary pattern score to all individuals within each quintile and treating this as a continuous variable. Generalized estimating equations (GEE) were used to account for census tract clustering. Potential confounding was addressed by building nested models to include demographic, socioeconomic, health and lifestyle factors identified a priori based on known associations with both diet and sleep (43–48). Models adjusted for: total energy intake, age, sex, race, education, employment, income category, number of people in house, spouse in the house, total population of census tract, ICE of census tract, smoking status, drinking status, caffeine intake, depressive symptoms, BMI, and non-work physical activity. Race was included in the model not based on hypothesized biological differences, but to capture some of the impact of centuries of structural racism and discrimination that has compounded towards a disproportionately high burden of adverse health outcomes for Black Americans. The inclusion of neighborhood-level factors also aimed to capture some of these effects. Interactions by sex, race, and education were tested for by including product terms in the adjusted model. Results were stratified if the coefficient for the product term was statistically significant at p < 0.05. Additional analyses included the following: adjusting for follow-up sleep duration in models with the insomnia outcome, removing BMI from the models with the sleep apnea outcome since the Berlin questionnaire includes BMI in its assessment, using the snoring and sleepiness components of the Berlin Questionnaire as outcomes, and using the components of the healthy sleep pattern as outcomes.

## Results

The mean age of the 593 included participants at the baseline (2001–2002) exam was 36 (SD: 4.4) years, 36% were men, and 30% were Black persons (Table 1). Nearly 60% reported annual household incomes of less than $45,000. The mean BMI was 29, 40% were people with obesity, 31% reported leisure time physical activity, and 31% had depressive symptoms (CES-D ≥ 16). The mean AHEI-2010 score at baseline was 39 (see **Table S4**, Additional file 1 for description of other dietary patterns). Those in higher quintiles of AHEI-2010 were older and more likely to be physically active in leisure time compared to those in lower quintiles (Table 1). At the 2013–2016 follow-up, 45% had insomnia symptoms, 41% had a high sleep apnea risk, and only 23% had a healthy sleep pattern. The proportion with insomnia symptoms was higher in the lowest quintile of AHEI compared to the highest quintile (57% vs. 32%, Table 1). Baseline characteristics by sleep outcomes at follow-up are reported in **Table S5** (Additional file 1).

We also compared the characteristics of those included in the study to other participants of the 2001–2002 BHS visit that were lost to follow-up or excluded (**Table S6**, Additional file 1). More men were lost or excluded, and they were more often current smokers and had a larger waist circumference compared to the included group. Differences across dietary patterns or sleep outcomes were minimal between these groups (p>0.05 for all comparisons).

There was a statistically significant inverse association between higher baseline diet quality measured by both AHEI-2010 and HEI-2015 and having fewer insomnia symptoms at follow-up (Table 2). These associations were significant in both unadjusted and fully adjusted models, accounting for socioeconomic, lifestyle, and health factors. After adjustment, each SD-increase in AHEI (SD=7.8 points) at baseline was associated with 15% lower risk of insomnia symptoms at follow-up, those in Q5 had 0.54 times the risk as those in Q1, and there was a significant decreasing risk trend across quintiles (RR [95% confidence interval (CI)]: per SD increase 0.85 [0.77, 0.93], Q5 vs. Q1: 0.54 [0.39, 0.75], p for trend: 0.0001). Similar results were seen using the HEI-2015 dietary pattern. In the fully adjusted model, participants in Q5 of HEI-2015 had 0.74 times the risk of insomnia symptoms at follow-up compared to those in Q1 (95% CI: 0.59, 0.92, p for trend: 0.001), and each SD-increase in HEI-2015 related to a 12% lower risk at follow-up (RR [95% CI]: 0.88 [0.82, 0.95]). There was no association between the aMed dietary pattern score and insomnia symptoms. In addition, no consistent effects were observed for having a high sleep apnea risk score or having a healthy sleep pattern at follow-up with any of the dietary patterns.

There were no meaningful interaction effects by sex for any of the associations tested. There were some statistically significant interactions observed by race (Black, White) and education status (Table 3). The association between baseline AHEI and insomnia symptoms at follow-up was modified by race (p for interaction 0.02). Upon stratification, White participants were less likely to have high risk for insomnia at follow-up if they had higher AHEI at baseline compared to white participants with lower AHEI scores (RR [95% CI]: Q5 vs. Q1: 0.47 [0.30, 0.74], per-SD increase: 0.84 [0.75, 0.94], p-trend: 0.0002), whereas no statistically significant effect was seen among Black participants. Education modified some of the associations between dietary patterns at baseline and having high sleep apnea score at follow-up. Higher diet quality measured by HEI-2015 and aMed was associated with lower risk of high sleep apnea score at follow up among those with education beyond a high school degree (RR [95% CI]: per SD increase in AHEI: 0.86 [0.77, 0.94], per SD increase in aMed: 0.92 [0.85, 0.99]), but this association was not observed among those with a high school degree or less (p for interaction for both HEI and aMed: 0.03).

Components of the AHEI-2010 score were evaluated individually for association with insomnia symptoms at follow-up (Table 4). Increased consumption of whole grains and long chain fatty acids were associated with lower risk of insomnia symptoms at follow-up (RR [95% CI] per one-unit increase in whole grain AHEI score: 0.91 [0.87, 0.95], long-chain (n-3) fats AHEI score: 0.95 [0.91, 0.98]). The opposite association was seen for the nuts and legumes component of AHEI-2010, which was associated with increased risk of insomnia symptoms (RR [95% CI] per one-unit increase in nuts and legumes AHEI score: 1.05 [1.02, 1.08]).

In sensitivity analysis, there was no difference in the association between diet quality and follow-up insomnia symptoms when sleep duration was added to the model (**Table S7**, Additional file 1). When BMI was removed from the model with sleep apnea risk as an outcome, the risk of having a high sleep apnea score at follow-up was lower for those in Q5 of AHEI compared to Q1 (RR [95% CI]: 0.68 [0.48, 0.97], **Table S8**, Additional file 1). There were also associations between higher AHEI at baseline and risk of being in the adverse group for the snoring and sleepiness components of the Berlin Questionnaire (**Table S9**, Additional file 1). Finally, when each component of the healthy sleep pattern was treated as the outcome, there were no associations except for the snoring component. Those with higher diet quality (AHEI-2010) had a higher risk of being in the healthy snoring group (no snoring reported in the previous 4 weeks) compared to those with lower AHEI scores (**Table S10**, Additional file 1).

## Discussion

This study found a higher diet quality, measured by AHEI-2010 and HEI-2015, in young adulthood was associated with lower risk of having insomnia symptoms at midlife, after an average of 12.7 years in the BHS cohort. This sample represents a lower-income, Black and White, semi-rural community in the southeastern U.S. After adjustment for several factors including multi-level socioeconomic status, physical activity, BMI, and depressive symptoms the RR for having insomnia symptoms at follow-up for those in Q5 compared to Q1 of baseline AHEI-2010 was 0.54. There were no statistically significant associations between young adult diet quality and sleep apnea risk or healthy sleep pattern at midlife.

This study adds to the sparse literature assessing prospective relationships between diet quality and future sleep outcomes. Castro-Diehl et al. found those with higher adherence to a Mediterranean diet were less likely to have concurrent short sleep and insomnia symptoms in the MESA cohort (16). In prospective analysis they found that those with an unchanged diet quality (aMed) over 10 years had fewer insomnia symptoms compared to those whose diet quality had decreased. In a prospective study among US women, Zuraikat et al. found higher adherence to the aMed diet associated with better sleep quality, higher sleep efficiency, and fewer sleep disturbances one year later (22). A third prospective study among European seniors identified lower odds of poor sleep quality and change in sleep duration (by 2 + hours) over 2.8 years of follow-up for those with higher Mediterranean diet adherence at baseline (21). Although our findings concur with these studies in that diet quality associates with less adverse sleep outcomes, a contrast is that we did not find any associations with the aMed diet. This could be due to population differences in dietary intake since the aMed is based on sex-specific median cut-offs of nine components. The score is influenced by total energy intake and the median intakes of aMed components within the specific population in which it is used. Those with higher energy intake will score highly if their intakes are above the population median value for most components and this is unlikely to be fully corrected for by adjusting for total energy. The aMed may also perform more poorly in a population with less variation in diet quality or a lower overall diet quality, as may be the case in a largely rural, lower income cohort with reduced access to healthy foods such as the BHS sample.

Diet may influence sleep through multiple pathways over both short and longer time frames. In the short term, intake of foods high in tryptophan, when combined with carbohydrate consumption and insulin release, appear to influence endogenous serotonin and melatonin synthesis, contributing to regulation of the sleep-wake cycle (49). Food intake triggers the release of numerous hormones, some of which can influence sleepiness, for example cholecystokinin which aids in breaking down proteins and fats, and may induce postprandial sleepiness (50, 51). Over longer time periods, diet quality can alter the microbiome, anthropometry, inflammation, and nutrient deficiencies which may all have impacts on sleep (52–55). A low-quality, energy-dense diet may lead to obesity which associates with a number of adverse sleep outcomes including insomnia (53, 54). Others have postulated that a higher quality diet has beneficial impacts on the gut microbiome which can in turn influence sleep quality and efficiency, as shown experimentally with consumption of probiotic-enriched fermented milk (9, 52). Nutrient deficiencies, such as inadequate vitamin D, may also play a role in increasing risk of sleep disorders (55).

We also tested for interaction effects and looked at the individual components of AHEI-2010. The association between higher diet quality and lower risk of insomnia was observed in White participants and among those with higher education, but not among Black participants or the less educated group. Although interpretation of these results should take into account the potentially limited power of subgroup analyses, they suggest that more marginalized populations experience additional burdens such that adhering to a higher diet quality is not sufficient to ward of insomnia, if the identified associations are causal.

This study has many strengths. First, diet was measured with a validated FFQ on average 13 years prior to sleep outcomes measured with validated questionnaires, allowing a prospective assessment of the impact of diet quality on future sleep. The BHS population enables expansion of the results from previous studies to a population more representative of a lower-income, non-urban community with a high proportion of Black people in the southeastern US, a region and demographic particularly impacted by health inequities. We controlled for many potential confounders, including neighborhood-level socioeconomic variables, physical activity, BMI, and depressive symptoms. Finally, the estimate of RRs instead of odds ratios (OR) is a strength since the sleep outcomes in this study were very common (40–45% prevalence at follow-up), so ORs would not approximate RRs. Despite that ORs are a valid effect measure regardless of their ability to approximate RRs, they are less intuitive and more prone to misinterpretation (56).

This study should be interpreted in light of the limitations. First, we did not have baseline sleep data so we could not exclude those with sleep apnea or insomnia symptoms at baseline nor could we assess change in sleep outcomes. It is not possible to know the direction or magnitude of the bias this caused, if any. However, these results are relevant regardless of this limitation since there have been very few prospective studies of this association to date and our study allows for a clear indexing of time. Second, we were unable to measure changes in diet across the follow-up time so we do not know if diet quality remained constant, improved, or worsened. We cannot infer the impact this might have had on the results with making large assumptions. As in all observational studies, we cannot eliminate the potential of residual confounding, including that caused by measurement error in covariates such as physical activity, measured here with self-report. However, this study controlled for several potential confounders identified a priori, including multi-level socioeconomic factors. Some effects may have gone undetected due to our pre-determined sample size, especially interaction effects and associations in stratified analyses. The testing of multiple dietary patterns, including the AHEI components, for associations with multiple sleep outcomes increased the number of tests performed which may increase the potential for type-1 error. However, all tests were planned *a priori*. Finally, the generalizability of the results is slightly altered from the original BHS cohort given that some differences were detected between those included versus those lost to follow-up or excluded.

## Conclusions

This study found that diet quality in young adulthood was inversely associated with insomnia symptoms in midlife. These findings need to be confirmed by additional, well-powered prospective studies which can account for baseline sleep. These results contribute evidence to understanding the role diet quality plays in sleep disorders over longer periods of time and may have implications for sleep- and diet-based interventions aiming to reduce chronic disease risk.

## Figures and Tables

**Table 1.** Description of participants in the total sample and by quintile of the Alternative Healthy Eating Index.

	Total sample ^a^	AHEI ^b^			p-value ^c^
		Q1	Q3	Q5	
	n=593	n=127	n=129	n=109	
***Demographic characteristics at baseline (2001-2002)***					
Age in years	36.31 ± 4.41	34.69 ± 4.63	36.18 ± 4.16	37.71 ± 4.21	<0.0001
Male (%)	36.26	35.43	32.56	39.45	0.420
Black persons (%)	30.02	23.62	33.33	37.61	0.131
Education, high school or less (%)	39.29	43.31	44.19	35.78	0.066
Income at baseline					
<$15,000	25.30	24.83	27.91	28.44	0.427
$15,000-$30,000	20.74	25.98	22.48	19.27	
$30,000-$45,000	12.82	16.54	13.95	9.17	
>$45,000	41.15	34.65	35.66	43.12	
Employed (%)	78.58	80.31	82.95	77.06	0.519
Has health insurance (%)	67.28	68.50	65.89	66.97	0.646
Household size	3.47 ± 1.31	3.60 ± 1.32	3.50 ± 1.36	3.32 ± 1.35	0.581
Lives with spouse (%)	61.89	65.35	60.47	52.29	0.113
Children in house (%)	73.69	74.80	75.19	71.56	0.854
***Neighborhood characteristics (census tract level, 2000 Decennial Census)***					
Total population	5070.39 ± 1848.51	5397.34 ± 1902.04	4894.37 ± 1736.07	4720.08 ± 1679.74	0.048
% of persons in poverty	23.66 ± 8.63	23.95 ± 7.09	23.48 ± 8.79	23.20 ± 9.22	0.819
Median household income	26670.86 ± 10397.49	25062.38 ± 5363.09	27147.77 ± 11391.39	27617.96 ± 11334.36	0.132
% of households with no vehicle	11.73 ± 6.90	10.91 ± 6.42	12.15 ± 6.89	11.92 ± 7.06	0.654
Index of Concentration at the Extremes	−0.13 ± 0.19	−0.13 ± 0.17	−0.13 ± 0.20	−0.13 ± 0.20	0.994
***Health and lifestyle factors at baseline (2001-2002)***					
Smoking status (%)					
Never	60.37	59.84	58.91	54.13	0.690
Former	12.65	10.24	12.40	16.51	
Current	26.98	29.92	28.68	29.36	
Current alcohol use (%)					
Non-drinker	35.41	44.88	33.33	20.18	<0.0001
Occasional drinker	39.12	39.37	45.74	32.11	
Regular drinker	25.46	15.75	20.93	47.71	
Total energy intake, kcal/d	2005.72 ±762.77	2134.99 ± 627.31	1972.44 ± 706.01	1936.93 ± 963.08	0.242
Caffeine intake, mg/d	98.38 ± 67.58	105.93 ± 65.91	96.59 ± 69.21	93.45 ± 68.43	0.555
Physically active at work (%)	39.63	40.94	42.64	39.45	0.151
Physically active not at work (%)	30.86	28.35	27.13	44.04	0.025
Depressive symptoms (%)	30.86	29.92	31.01	33.94	0.877
CES-D score	12.61 ± 8.91	12.51 ± 9.34	12.42 ± 8.18	13.35 ± 9.62	0.874
Body mass index, kg/m^2^	29.25 ± 7.27	30.14 ± 7.56	28.57 ± 7.01	28.85 ± 7.21	0.239
Obesity (%)	39.63	45.67	34.88	33.94	0.199
Waist circumference, cm	92.42 ± 17.03	95.36 ± 17.24	90.39 ± 16.40	91.72 ± 17.39	0.079
***Sleep outcomes at follow-up (2013-2016)***					
High risk for insomnia (%)	45.19	57.48	48.84	32.11	0.002
High risk for sleep apnea (%)	40.81	46.36	40.31	34.86	0.510
Healthy sleep pattern (%) *	22.66	20.00	21.71	23.36	0.851

a. Frequency (%) or mean ±SD among total sample.

b. Frequency (%) or mean ±SD among column total (total in quintile).

c. p-value from ANOVA for continuous covariates and from Pearson chi-squared test for independence for categorical covariates, testing for differences across all 5 quintiles (Q2 and Q4 not shown).

* n=589 for Healthy Sleep Pattern

AHEI: Alternative Healthy Eating Index.

Physically active: self-rating of 4 or 5 (active or very active) on 5-point scale.

Index of Concentration at the Extremes: ([(number of White householders with >$100,000 annual income)-(number of Black householders with <$25,000 annual income)] / total households reporting income)

Occasional drinker: less than once a week; Regular drinker: once or twice a week, or more.

Depressive symptoms defined as CES-D ≥ 16.

Obesity defined as BMI ≥ 30 kg/m^2^.

High risk for insomnia: score >9 on the Women’s Health Initiative Insomnia Rating Scale.

High risk for sleep apnea: positive on two of three categories on the Berlin questionnaire.

Healthy sleep pattern: morning or more morning-than-evening chronotype, average sleep duration ≥7 and <9 hours, insomnia symptoms <1-2 times/wk, snoring is quiet and infrequent (≤1-2 times/wk), and no excessive daytime sleepiness.

**Table 2.** Risk ratios for sleep outcomes at follow-up by baseline dietary pattern scores.

	model	Quintile of dietary pattern score at baseline (2001-2002)					p for trend	per SD increase in dietary pattern ^a^
		Q1	Q2 ^a^	Q3 ^a^	Q4 ^a^	Q5 ^a^		
**AHEI 2010**								
Q n (median)		127 (28.66)	116 (33.93)	129 (37.57)	112 (42.41)	109 (48.99)		sd=7.8
Insomnia risk	1	1.00	0.72 (0.56, 0.93) *	0.85 (0.74, 0.98) *	0.76 (0.65, 0.90) ***	0.56 (0.42, 0.74) ***	<0.0001	0.86 (0.80, 0.92) ***
	2	1.00	0.74 (0.59, 0.93) **	0.84 (0.73, 0.97) *	0.77 (0.65, 0.90) **	0.56 (0.43, 0.74) ***	<0.0001	0.86 (0.79, 0.93) ***
	3	1.00	0.75 (0.60, 0.93) *	0.83 (0.69, 1.00)	0.76 (0.63, 0.91) **	0.54 (0.39, 0.75) ***	0.0001	0.85 (0.77, 0.93) ***
Sleep apnea risk	1	1.00	0.87 (0.73, 1.04)	0.87 (0.64, 1.17)	0.88 (0.68, 1.14)	0.75 (0.48, 1.16)	0.200	0.93 (0.82, 1.05)
	2	1.00	0.85 (0.69, 1.03)	0.83 (0.60, 1.14)	0.84 (0.64, 1.11)	0.68 (0.42, 1.12)	0.134	0.90 (0.78, 1.04)
	3	1.00	0.86 (0.68, 1.08)	0.90 (0.61, 1.32)	0.90 (0.66, 1.23)	0.70 (0.41, 1.21)	0.225	0.92 (0.79, 1.06)
Healthy sleep pattern	1	1.00	1.13 (0.83, 1.55)	1.09 (0.71, 1.66)	1.31 (0.96, 1.78)	1.17 (0.76, 1.79)	0.277	1.04 (0.94, 1.14)
	2	1.00	1.09 (0.76, 1.56)	1.09 (0.69, 1.72)	1.27 (0.95, 1.70)	1.18 (0.69, 2.03)	0.367	1.05 (0.92, 1.19)
	3	1.00	1.07 (0.73, 1.58)	1.00 (0.58, 1.70)	1.23 (0.91, 1.66)	1.18 (0.69, 2.01)	0.327	1.05 (0.94, 1.18)
**HEI 2015**								
Q n (median)		111 (45.89)	116 (52.62)	130 (57.47)	114 (62.58)	122 (69.98)		sd=9.4
Insomnia risk	1	1.00	1.13 (0.96, 1.32)	0.88 (0.71, 1.10)	0.78 (0.51, 1.20)	0.70 (0.55, 0.88) **	0.003	0.87 (0.80, 0.95) **
	2	1.00	1.14 (0.97, 1.34)	0.88 (0.71, 1.08)	0.77 (0.51, 1.15)	0.71 (0.57, 0.88) **	0.0002	0.87 (0.81, 0.94) ***
	3	1.00	1.11 (0.93, 1.33)	0.89 (0.72, 1.10)	0.77 (0.52, 1.15)	0.74 (0.59, 0.92) **	0.001	0.88 (0.82, 0.95) **
Sleep apnea risk	1	1.00	1.19 (0.95, 1.49)	1.29 (1.03, 1.62) *	1.12 (0.81, 1.53)	0.91 (0.68, 1.23)	0.439	0.94 (0.85, 1.03)
	2	1.00	1.15 (0.91, 1.44)	1.22 (0.94, 1.58)	1.06 (0.76, 1.48)	0.85 (0.61, 1.18)	0.276	0.91 (0.82, 1.01)
	3	1.00	1.14 (0.92, 1.41)	1.29 (0.99, 1.69)	1.08 (0.75, 1.55)	0.89 (0.63, 1.25)	0.459	0.93 (0.83, 1.04)
Healthy sleep pattern	1	1.00	1.19 (0.61, 2.32)	0.80 (0.49, 1.29)	1.03 (0.62, 1.71)	1.04 (0.68, 1.58)	0.914	0.98 (0.86, 1.12)
	2	1.00	1.32 (0.68, 2.57)	0.91 (0.52, 1.59)	1.18 (0.74, 1.90)	1.15 (0.68, 1.95)	0.708	1.01 (0.87, 1.17)
	3	1.00	1.40 (0.73, 2.70)	0.83 (0.48, 1.43)	1.15 (0.68, 1.94)	1.05 (0.63, 1.74)	0.943	0.97 (0.84, 1.11)
**aMed**								
Q n (median)		98 (2.00)	90 (3.00)	121 (4.00)	127 (5.00)	157 (6.00)		sd=1.8
Insomnia risk	1	1.00	0.79 (0.63, 0.99) *	0.86 (0.71, 1.04)	0.79 (0.62, 1.00) *	0.91 (0.68, 1.20)	0.605	0.96 (0.88, 1.05)
	2	1.00	0.81 (0.66, 0.99) *	0.88 (0.73, 1.04)	0.83 (0.65, 1.07)	0.93 (0.70, 1.23)	0.768	0.97 (0.88, 1.06)
	3	1.00	0.80 (0.67, 0.97) *	0.88 (0.73, 1.06)	0.85 (0.64, 1.11)	0.95 (0.70, 1.28)	0.926	0.98 (0.88, 1.09)
Sleep apnea risk	1	1.00	1.03 (0.74, 1.43)	1.14 (0.81, 1.59)	1.19 (0.92, 1.53)	1.03 (0.79, 1.33)	0.565	1.02 (0.95, 1.08)
	2	1.00	1.02 (0.75, 1.39)	1.07 (0.79, 1.45)	1.08 (0.86, 1.35)	0.92 (0.69, 1.22)	0.597	0.97 (0.89, 1.05)
	3	1.00	1.05 (0.75, 1.46)	1.11 (0.82, 1.52)	1.17 (0.91, 1.49)	0.96 (0.72, 1.28)	0.880	0.99 (0.90, 1.08)
Healthy sleep pattern	1	1.00	1.13 (0.74, 1.74)	0.86 (0.58, 1.26)	0.72 (0.55, 0.94) *	0.83 (0.59, 1.18)	0.023	0.90 (0.81, 1.01)
	2	1.00	1.13 (0.71, 1.79)	0.90 (0.58, 1.39)	0.79 (0.60, 1.06)	1.03 (0.64, 1.66)	0.661	0.98 (0.84, 1.15)
	3	1.00	1.15 (0.78, 1.71)	0.88 (0.62, 1.25)	0.77 (0.59, 0.99) *	1.01 (0.64, 1.60)	0.519	0.97 (0.84, 1.12)

a. Risk Ratio (95% Confidence Interval)

* p < 0.05

** p < 0.01

*** p < 0.001

Model 1: unadjusted.

Model 2: total energy intake, age, sex, race, education (any college or higher), employed (full or part time), income category, number of people in house, spouse lives in house, total population of census tract, ICE of census tract.

Model 3: model 2 + smoking status (never, current, former), drinking status (non-, occasional, regular drinker), caffeine intake (mg/d), depressive symptoms (CES-D≥16), body mass index (kg/m^2^), physical active when not at work (4 or 5 [active or very active] on 5-point self-rating scale).

**Table 3.** Risk ratios for sleep outcomes by baseline dietary pattern scores by race/ethnicity and education.

	model	Quintile of dietary pattern score at baseline (2001-2002)					p for trend	per SD increase ^a^	p interaction ^b^
		Q1	Q2 ^a^	Q3 ^a^	Q4 ^a^	Q5 ^a^			
**Race stratified** (n Black = 178, n White = 415)									
**AHEI 2010**									
Insomnia risk	Black	1.00	0.50 (0.36, 0.71) ***	0.77 (0.55, 1.06)	0.76 (0.46, 1.27)	0.69 (0.43, 1.08)	0.506	0.95 (0.77, 1.16)	0.023
	White	1.00	0.82 (0.65, 1.03)	0.86 (0.69, 1.06)	0.78 (0.65, 0.94) **	0.47 (0.30, 0.74) **	0.0002	0.84 (0.75, 0.94) **	
**Education stratified** (n low = 233, n high = 360)									
**HEI 2015**									
Sleep apnea risk	low	1.00	1.34 (0.95, 1.89)	1.66 (1.08, 2.55) *	1.72 (1.06, 2.80) *	1.08 (0.64, 1.82)	0.243	1.09 (0.91, 1.31)	0.030
	high	1.00	0.97 (0.64, 1.48)	1.05 (0.77, 1.43)	0.79 (0.51, 1.21)	0.73 (0.49, 1.09)	0.037	0.86 (0.77, 0.95) **	
**aMed**									
Sleep apnea risk	low	1.00	1.31 (0.62, 2.79)	1.57 (0.89, 2.76)	1.67 (0.85, 3.31)	1.34 (0.72, 2.49)	0.238	1.12 (0.91, 1.38)	0.030
	high	1.00	0.92 (0.64, 1.32)	0.91 (0.66, 1.25)	0.92 (0.70, 1.21)	0.78 (0.62, 0.98) *	0.050	0.92 (0.85, 0.99) *	

a. Risk Ratio (95% Confidence Interval)

b. p for interaction between the dietary pattern trend variable and the effect modifier of interest (race or education).

* p < 0.05

** p < 0.01

*** p < 0.001

Models adjusted for: total energy intake, age, sex, race, education (any college or higher), employed (full or part time), income category, number of people in house, spouse lives in house, total population of census tract, ICE of census tract, smoking status (never, current, former), drinking status (non-, occasional, regular drinker), caffeine intake (mg/d), depressive symptoms (CES-D≥16), body mass index (kg/m^2^), physical active when not at work (4 or 5 [active or very active] on 5-point self-rating scale).

**Table 4.** Risk ratios for insomnia by baseline scores of components of the Alternate Healthy Eating Index.

	**Quintile of AHEI-2010 component score at baseline (2001-2002)**						
Component of AHEI-2010 dietary pattern	Q1	Q2 ^a^	Q3 ^a^	Q4 ^a^	Q5 ^a^	p for trend	per one unit increase ^a^
1. Fruits	1.00	1.02 (0.77, 1.34)	1.04 (0.80, 1.35)	1.14 (0.98, 1.34)	0.80 (0.61, 1.04)	0.082	0.97 (0.93, 1.01)
2. Vegetables (not potatoes)	1.00	0.97 (0.77, 1.23)	0.96 (0.79, 1.17)	0.97 (0.77, 1.24)	0.80 (0.56, 1.15)	0.176	0.96 (0.92, 1.01)
3. Nuts and legumes	1.00	1.27 (1.01, 1.60) *	1.22 (0.92, 1.61)	1.51 (1.14, 2.00) **	1.63 (1.31, 2.04) ***	<0.0001	1.05 (1.02, 1.08) ***
4. Whole grains	1.00	0.87 (0.74, 1.02)	0.74 (0.58, 0.96) *	0.81 (0.65, 1.01)	0.56 (0.41, 0.77) ***	0.003	0.91 (0.87, 0.95) ***
5. Long chain (n-3) fats	1.00	1.11 (0.89, 1.38)	0.87 (0.67, 1.13)	1.06 (0.90, 1.25)	0.73 (0.58, 0.93) **	0.004	0.95 (0.91, 0.98) **
6. Polyunsaturated fats	1.00	0.85 (0.64, 1.13)	0.89 (0.66, 1.19)	0.89 (0.67, 1.18)	0.83 (0.64, 1.07)	0.204	0.96 (0.91, 1.01)
7. Sugar sweetened beverages and fruit juice ^b^	1.00	0.86 (0.58, 1.27)	0.79 (0.62, 1.01)	NA	NA	0.069	0.97 (0.93, 1.00)
8. Red and processed meats	1.00	0.87 (0.68, 1.11)	0.97 (0.75, 1.27)	0.89 (0.61, 1.30)	0.80 ().54, 1.19)	0.371	0.98 (0.94, 1.03)
9. Sodium	1.00	1.08 (0.86, 1.36)	1.03 (0.80, 1.31)	0.97 (0.70, 1.34)	1.15 (0.88, 1.51)	0.435	1.02 (0.99, 1.05)
10. Alcohol ^b^	1.00	0.82 (0.48, 1.38)	0.66 (0.45, 0.97) *	0.76 (0.50, 1.16)	NA	0.213	0.98 (0.96, 1.01)

a. Risk Ratio (95% confidence interval)

b. There are only three groups (tertiles) for sugar sweetened beverages and four groups (quartiles) for alcohol.

* p < 0.05

** p < 0.01

*** p < 0.001

Models adjusted for: total energy intake, age, sex, race, education (any college or higher), employed (full or part time), income category, number of people in house, spouse lives in house, total population of census tract, ICE of census tract, smoking status (never, current, former), drinking status (non-, occasional, regular drinker), caffeine intake (mg/d), depressive symptoms (CES-D≥16), body mass index (kg/m^2^), physical active when not at work (4 or 5 [active or very active] on 5-point self-rating scale).

## Data Availability

The datasets used and analyzed during the current study are available from the corresponding author on reasonable request.

## Abbreviations

AHEI: Alternate Healthy Eating Index
aMed: Alternate Mediterranean Dietary Pattern
BHS: Bogalusa Heart Study
CESD: Center for Epidemiologic Studies Depression Scale
Delta NIRI: Lower Mississippi Delta Nutrition Intervention Research Initiative Food Frequency Questionnaire
GEE: Generalized Estimating Equations
HEI: Healthy Eating Index
ICE: Index of Concentration at the Extremes
IPAQ: International Physical Activity Questionnaire
MESA: Multi-Ethnic Study of Atherosclerosis
MET: Metabolic Equivalent of Task
mRFEI: Modified Retail Food Environment Index
PRR: Prevalence Rate Ratio
Q: Quintile
TEI: Total Energy Intake
WHIIRS: Women’s Health Initiative Insomnia Rating Scale
